# Retraction: Role of Vitamin-D Receptor (VDR) single nucleotide polymorphisms in gestational hypertension development: A case-control study

**DOI:** 10.1371/journal.pone.0339144

**Published:** 2025-12-18

**Authors:** The PLOS One Editors

The *PLOS One* Editors retract this article [1] because it was identified as one of a series of submissions for which we have concerns about compromised peer review and compliance with PLOS policy on Manipulation of the Publication Process.

All authors either did not respond directly or could not be reached.
